# Synthesis Pathway of Layered-Oxide Cathode Materials for Lithium-Ion Batteries by Spray Pyrolysis

**DOI:** 10.1021/acsami.4c06503

**Published:** 2024-06-24

**Authors:** Manar Almazrouei, Sulki Park, Maurits Houck, Michael De Volder, Simone Hochgreb, Adam Boies

**Affiliations:** † Department of Engineering, University of Cambridge, Cambridge CB2 1PZ, United Kingdom; ‡ Department of Mechanical and Aerospace Engineering, United Arab Emirates University, Al Ain 15551, Abu Dhabi, United Arab Emirates; ¶ Echion Technologies, Ltd., Sawston, Cambridge CB22 3FG, United Kingdom

**Keywords:** lithium cobalt oxide, spray pyrolysis, structure property relationship, annealing conditions, lithium-ion battery

## Abstract

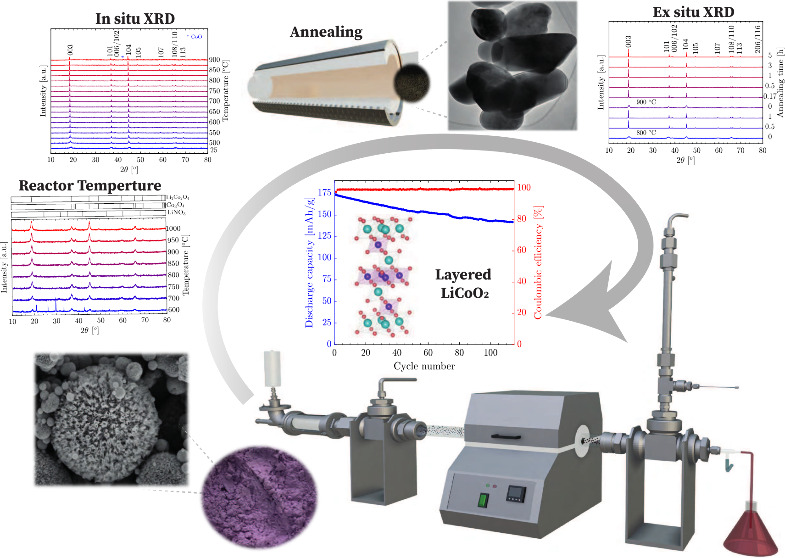

We report the synthesis of LiCoO_2_ (LCO) cathode materials for lithium-ion batteries via aerosol spray pyrolysis, focusing on the effect of synthesis temperatures from 600 to 1000 °C on the materials’ structural and morphological features. Utilizing both nitrate and acetate metal precursors, we conducted a comprehensive analysis of material properties through X-ray diffraction (XRD), Raman spectroscopy, thermogravimetric analysis (TGA), and scanning electron microscopy (SEM). Our findings reveal enhanced crystallinity and significant oxide decomposition within the examined temperature range. Morphologically, nitrate-derived particles exhibited hollow, spherical shapes, whereas acetate-derived particles were irregular. Guided by high-temperature X-ray diffraction (HT-XRD) data, the formation of a layered LCO oxide structure, with distinct spinel Li_2_Co_2_O_4_ and layered oxide LCO phases was shown to emerge at different annealing temperatures. Optimally annealed particles showcased well-defined layered structures, translating to high electrochemical performance. Specifically, nitrate-based particles annealed at 775 °C for 1 h demonstrated initial discharge capacities close to 179 mAh/g, while acetate-based particles, annealed at 750 °C for 3 h, achieved 136 mAh/g at a 0.1*C* discharge rate. This study elucidates the influence of synthesis conditions on LCO cathode material properties, offering insights that advance high throughput processes for lithium-ion battery materials synthesis.

## Introduction

Lithium-ion batteries (LIBs) stand at the forefront of energy storage technology, powering a vast range of applications from electronic devices to electric vehicles (EVs) and grid storage systems. Since the first commercialization by SONY, cobalt (Co) has been used in cathode materials, such as LiCoO_2_ (LCO).^1^ While work has hence sought to reduce Co content in cathodes due to its toxicity and high cost, Co remains prevalent in LIBs, especially for portable electronics. It remains in new formulations of LiNi_*x*_Co_*y*_Mn_*z*_O_2_ (NMC) cathode materials, and coatings, which are favored in EV applications.^2^

The synthesis of LIB cathode materials has been achieved through various methods including coprecipitation, solid-state, sol–gel, hydrothermal, spray pyrolysis (SP), and combustion techniques. Coprecipitation is a popular synthesis method due to its homogeneous mixing at the atomic scale and particle morphology control. Despite its advantages, coprecipitation involves a multistep process that includes mixing, precipitation, purification, lithiation, and sintering, which cumulatively elevate the cost of production.^3^ Coprecipitation requires an inert atmosphere to prevent impurity formation and precise control over numerous parameters, including pH, concentration, precipitation/reaction time, temperature, and stirring rate, which influence particle size and morphology. The process also generates a large volume of ion-containing liquid waste and nitrogen-containing chemical waste due to the evaporation and oxidation of ammonia in aqueous solutions.^4^ In contrast, SP offers a scalable, continuous process that can achieve homogeneous compositions within seconds. SP enables rapid and homogeneous material synthesis, which can be crucial for achieving high throughput and efficiency, especially in large-scale manufacturing environments. SP is favored for its simplicity, cost-effectiveness, reduced process steps, and relatively environmentally friendly nature. Additionally, SP allows for precise control over composition, which can lead to tailored material properties suitable for various applications. However, SP also faces challenges that require further investigation and optimization. One of the main issues is the formation of hollow shell-like particles due to solute concentration gradients during solvent evaporation, which can result in low product density. The collection of synthesized aerosol particles is another aspect that may require optimization to ensure efficient recovery of the product.^3^

Despite SP’s utilization of high-temperature tube furnaces, additional heat treatment is essential for obtaining high-purity, crystalline material. Determining the optimal annealing temperature and duration typically involves extensive experimentation, being both time-consuming and costly. Various factors like synthesis method, precursor materials, and lithium source critically influence these conditions. Researchers have explored the effects of synthesis and sintering temperatures on cathode properties and electrochemical performance. For instance, Choi et al.^5^ investigated the optimal synthesis and sintering conditions for LCO synthesized by SP through a statistical experimental design method. Similarly, Habibi et al.^6^ conducted numerous experiments to study the effects of sintering conditions on NMC111 particle crystal growth and electrochemical performance synthesized by the solution combustion process. Likewise, Ju et al.^7^ studied the influence of various annealing temperatures on NMC111 particles produced by the SP technique while maintaining a fixed reactor/synthesis temperature and annealing time. Lower annealing temperature can result in a poor layered hexagonal structure, and a higher temperature can lead to lithium evaporation. The annealing conditions can vary between various synthesis techniques despite the similar composition.

**Figure 1:**
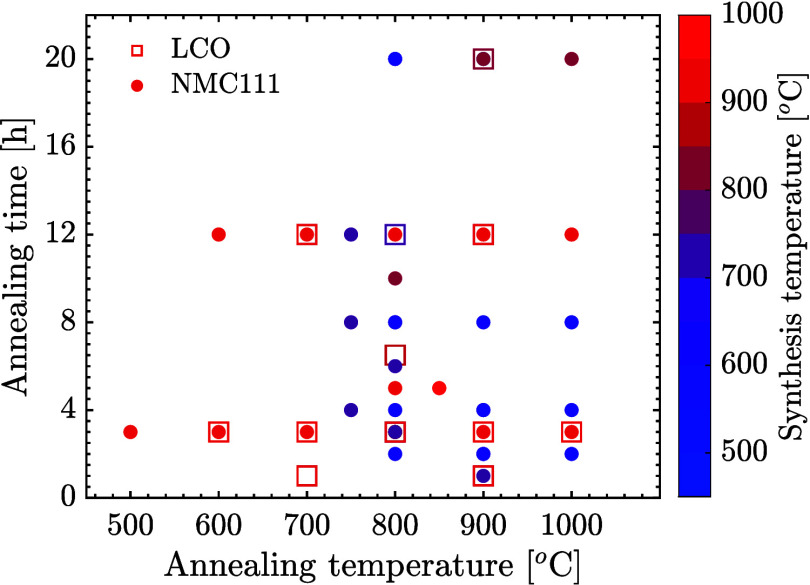
Comparison of synthesis and annealing conditions for LCO and NMC111 cathode materials using the SP technique, as reported in the literature.^5,7−27^

Figure 1 offers a thorough overview of the reported synthesis temperatures and annealing conditions (both temperature and time) for LCO and NMC111 particles synthesized via the SP method. Both materials share similar structures and properties. The reported reactor temperature for both LCO and NMC111 falls within the range of 450–1000 °C, while the annealing temperature and duration range from 500 to 1000 °C and 1–20 h, respectively.

Understanding the structural transformations of LCO is crucial, as it can exist in two primary forms with distinct electrochemical properties: the low-temperature spinel Li_2_Co_2_O_4_ (LT-LCO) and the high-temperature layered LiCoO_2_ (HT-LCO). LCO exhibits two distinct crystallographic structures based on synthesis temperature, adopting a spinel structure at lower temperatures (LT-LCO) and transitioning to a layered trigonal (or hexagonal) structure at higher temperatures (>750 °C, HT-LCO). The spinel Li_2_Co_2_O_4_ has a cubic structure (space group *Fd*3̅*m*) characterized by a framework of mixed Li and Co cations. This intermixing of cations disrupts the lithium diffusion pathways, leading to poor lithium mobility and limited electrochemical performance. As a result, LT-LCO displays poor cycling performance, making it unfavorable as a cathode material. In contrast, layered LiCoO_2_ crystallizes in a hexagonal structure (space group *R*3̅*m*) with alternating layers of lithium and cobalt ions. This well-ordered arrangement provides distinct two-dimensional lithium diffusion channels parallel to the layers, enabling facile lithium intercalation and deintercalation. As a result, layered LiCoO_2_ exhibits superior electrochemical properties compared to the spinel phase.^2,28,29^

Duffiet et al.^2^ and Gim et al.^30^ investigated LCO crystallization from solid-state reactions using HT-XRD. However, no study has yet delved into the structural evolution of LCO synthesized via SP or used HT-XRD to optimize annealing conditions for SP-synthesized LCO. Acetate precursors, while less commonly adopted, offer advantages in terms of reduced toxicity and ease of handling; however, they are also known to potentially introduce carbonate impurities, a drawback not typically associated with nitrate precursors, which are favored for their higher solubility and capability to yield products of superior purity.^20,31^ Although nitrates and acetates have been explored separately as precursors, no direct comparison of their viability has been made.

This study aims to bridge these knowledge gaps by investigating how synthesis conditions affect the structure and morphology of LCO cathode materials derived from nitrate and acetate precursors via SP. It utilizes HT-XRD analyses to study the structural evolution of synthesized particles at varying synthesis temperatures and selects optimal annealing conditions from these analyses, thereby eliminating extensive experimentation. Furthermore, it examines the impact of the chosen temperatures on the structure, properties, and electrochemical performance of ex-situ LCO samples.

## Experimental Section

### Synthesis of LiCoO_2_ via Spray Pyrolysis Reactor

LCO was synthesized employing the SP technique, utilizing both nitrate and acetate-based precursors. The nitrate precursor was formulated by dissolving cobalt nitrate hexahydrate [Co(NO_3_)_2_·6H_2_O, Thermo Scientific] and lithium nitrate [LiNO_3_, Thermo Scientific] in deionized water, achieving a Li:Co molar ratio of 1.05:1. The solution concentration was maintained at 1 mol/L, with an additional 5% stoichiometry to offset lithium loss during the synthesis process. In parallel, the acetate precursor was prepared by dissolving cobalt(II) acetate tetrahydrate [C_4_H_6_CoO_4_·4H_2_O, Acros Organics] and lithium acetate dihydrate [C_2_H_3_LiO_2_·2H_2_O, Acros Organics] in deionized water, ensuring the same metal ratios, concentration, and excess lithium were preserved.

**Figure 2:**
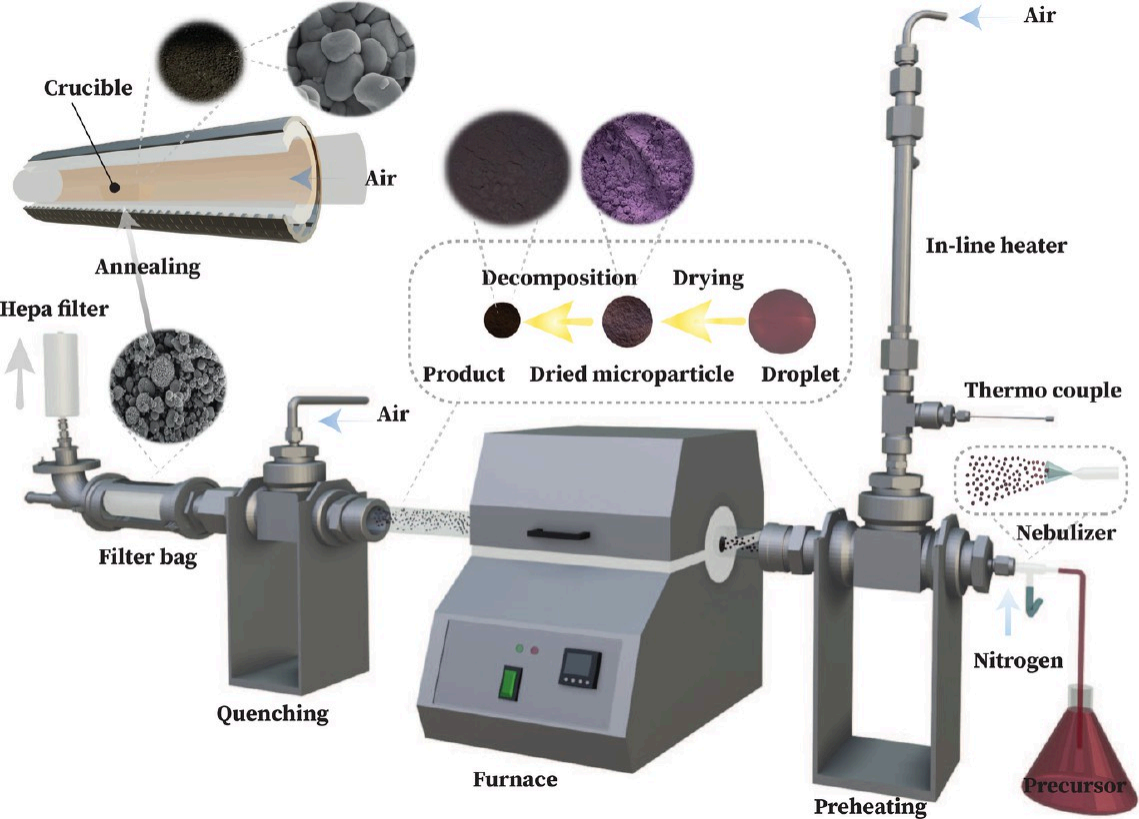
Schematic representation of the spray pyrolysis reactor setup for synthesizing LiCoO_2_.

The synthesis system comprised a preheating segment, a high-temperature furnace, a quenching module, and a collection mechanism. The configuration of the spray pyrolysis apparatus is illustrated in Figure 2. A quartz nebulizer atomized the precursor solutions into fine aerosol droplets, subsequently vaporized in a stainless steel preheating section. This section operated at an uptake rate of 1 m/min and was coupled with a nitrogen flow rate of 1 L/min (99.998% purity, BOC), with the preheating temperature regulated at 400 °C to ensure thorough vaporization. The average temperature achieved in this section was ∼230 °C. Droplet size distribution, characterized by a Sauter mean diameter ranging from 21 to 61 μm, was determined using Dantec Dynamics Phase Doppler Anemometry (Figure S1).

Postvaporization, the dried droplets were directed into a tube furnace (quartz tube). The heated section of the tube furnace was set to a temperature range of 600–1000 °C, which sets peak temperature in a parabolic axial furnace distribution. The temperature was varied to determine the impact on the particles’ morphology and structure. The quenching section, employing an air flow rate of 30 L/min, reduced the temperature of the decomposed particles, which had partially transformed into oxides (discussed further below), to below 150 °C before collecting them in a filter bag. To prevent particle emission into the environment, a HEPA filter was installed at the end of the filter bag housing. For the purpose of achieving a higher crystalline layered structure, the collected particles (approximately 2 g) with promising structure and morphology were annealed in an alumina crucible. They were placed inside a second tube furnace with a 1 L/min airflow. The annealing temperature was determined based on the HT-XRD results, using a ramping rate of 5 °C/min (see materials characterization techniques in the Supporting Information).

### Coin Cell Assembly and Electrochemical Tests

The slurry for nitrate-derived LCO was formulated by combining the active material with carbon black (Super-P) and polyvinylidene fluoride (PVDF) in a weight ratio of 90:5:5. PVDF was dissolved in *N*-methyl-2-pyrrolidone (NMP) to fine-tune the slurry’s viscosity, ensuring optimal electrode coating quality. The mixture was blended at 2000 rpm in a Thinky mixer, cast onto aluminum foil using a linear coater set to a 150 μm gap, and dried at 120 °C for 30 min. The electrodes, shaped into 13 mm discs, were dried overnight in a vacuum oven at the same temperature. CR2032 coin cells were assembled in an argon-filled glovebox, each containing a 15 mm Li metal disc counter electrode, a 19 mm diameter Celgard separator, and filled with 20 μL of LP40 electrolyte (1 M LiPF_6_ in EC/DEC, 1:1 v/v). Cells were crimped at 1000 psi and underwent electrochemical testing, including formation cycling at 0.1*C* (*C* = 160 mAh/g) and stability testing at 0.5*C* within a 3–4.3 V voltage window, employing a Biologic BCS 805 Series instrument.

Adjusting for the acetate-derived LCO’s porous aggregated structure, the electrode composition was optimized to an 80:10:10 ratio of LCO to Super P to PVDF. This slurry was mixed, cast, dried, and calendared to enhance the electrode’s structural integrity and ensure effective electrolyte infiltration. The processed electrodes were punched into 14 mm discs and assembled into 2032-type coin cells, incorporating an 18 mm Celgard separator and 80 μL of 1.3 M LiPF_6_ electrolyte in EC:DEC (3:7 wt/wt), tailored for the electrode’s specific needs. Crimped at 700 psi, the cells were conditioned for 8 h before being subjected to formation cycling at 0.1*C*, stability tests at 0.5*C*, and rate capability assessments ranging from 0.5*C* to 20*C*. Testing was conducted in a controlled environment at 25 °C using Biologic VSP.

## Results and Discussion

### Effect of Synthesis Conditions

The physicochemical attributes of particles synthesized via SP are intricately linked to the synthesis conditions employed. These parameters (encompassing the type and concentration of the precursor, reactor temperature, flow rate, and residence time) play a pivotal role in determining the final properties of the synthesized materials.^32^ Nitrate-based precursors are the conventional choice for the synthesis of cathode materials such as LCO and NMC111, frequently utilized in conjunction with separate lithium sources (e.g., LiOH) or mixed with other metals within the same precursor solution, including LiNO_3_ and Li_2_CO_3_.^5,9,12^

**Figure 3:**
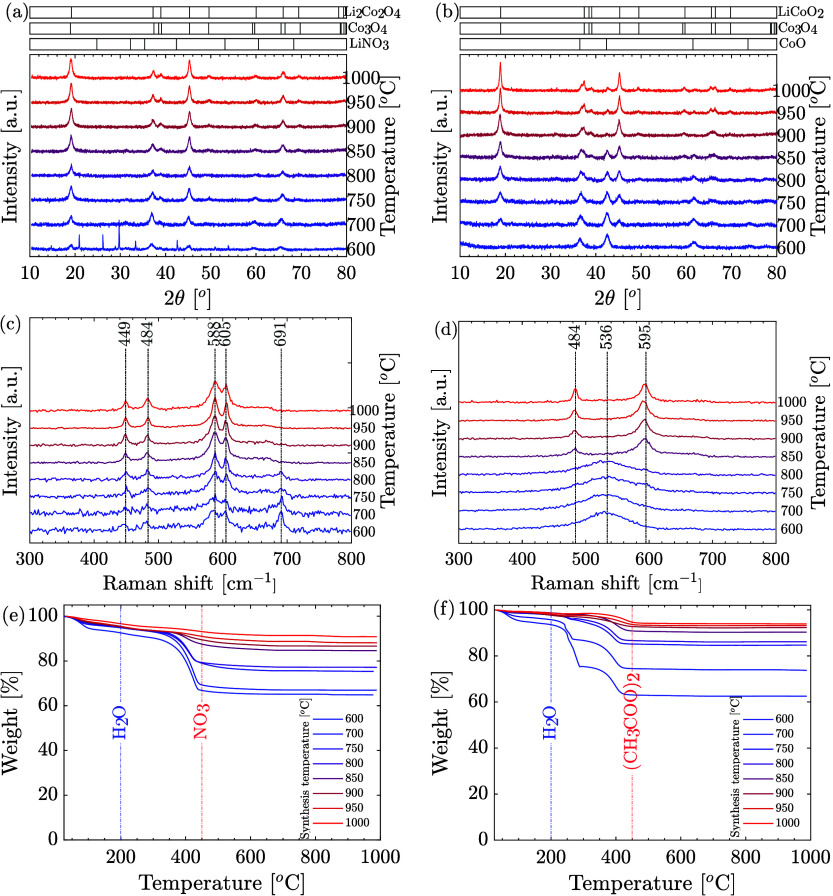
Characterization of as-synthesized LCO particles under varying reactor temperatures using nitrate (left) and acetate (right) as metals and lithium sources. (a, b) XRD patterns with reference positions of existing phases, (c, d) Raman spectra, and (e, f) TGA analysis, highlighting the impact of synthesis conditions on particle physicochemical properties.

This investigation spans synthesis temperatures, from 600 to 1000 °C, with a controlled preheating zone temperature of ∼230 °C and maintaining a consistent residence time of ∼0.6 s in ambient conditions (25 °C) within the heated section of the electric furnace. The impact of these synthesis parameters on the physicochemical properties of the as-synthesized particles was examined using XRD, Raman spectroscopy, and TGA (Figure 3).

XRD depicts the phase evolution of LCO particles synthesized via SP from metal and lithium nitrate precursors, as illustrated in Figure 3a. Initial observations revealed that particles synthesized at lower temperatures (600 and 700 °C) were hygroscopic and exhibited phase instability under ambient conditions, with distinct peaks for unreacted nitrate evident at 600 °C. These findings align with the documented ICSD code 67981 for LiNO_3_, highlighting the presence of undecomposed nitrate. As the synthesis temperature increased, a noticeable reduction in undecomposed nitrate was observed, giving rise to a Co_3_O_4_ spinel phase (ICSD code 24210) characterized by enhanced crystallinity, as indicated by the emergence of sharper and more intense peaks at temperatures of 700 °C and above. This phase evolution suggests the formation of a partially lithiated Co_3_O_4_ structure, potentially transitioning to a low-temperature LT-LCO phase (ICSD code 74320), dependent on the lithium source’s melting point, notably 253 °C for LiNO_3_.^2,31^

The phase diagram (see Figure S2) underscores the Co_3_O_4_ phase’s formation in an oxygen-rich environment up to 1000 °C.^33^ XPS results, discussed in detail in the “Effect of Annealing Conditions” section, confirm the presence of lithium in the form of Li_2_O_2_ within the synthesized particles. Given the challenges associated with detecting amorphous or poorly crystalline phases via XRD due to the technique’s limited sensitivity, Raman spectroscopy was employed to further determine the phase composition of the nitrate-derived particles (Figure 3c). The Raman spectra revealed a distinct band at 691 cm^–1^ (*A*_1*g*_) indicative of Co_3_O_4_, persisting up to 800 °C, alongside bands characteristic of the LT-LCO phase, confirming lithiation at temperatures exceeding 850 °C.^2,34,35^

Conversely, the XRD analysis of particles synthesized from acetate precursors (Figure 3b) demonstrated an absence of undecomposed metal or lithium precursors at 600 °C, with the phase diagram suggesting the formation of rock-salt CoO (ICSD code 9865). As temperatures increased to 700 °C and beyond, Co_3_O_4_ became more prominent, as shown by distinct peak shifts and a decrease in CoO signals. At 900 °C, Co_3_O_4_ became the dominant phase, accompanied by the emergence of additional characteristic peaks.

To mitigate misinterpretations of XRD spectra, including Li_2_CO_3_ in the precursor (see the XPS results shown in the Supporting Information), Raman spectroscopy served as an essential tool for detailed phase analysis. The Raman spectra of particles synthesized within the 600–800 °C temperature range revealed a broad band between 524 and 536 cm^–1^, indicative of the coexistence of CoO and Co_3_O_4_ phases, corroborating XRD results.^36^ Notably, at elevated synthesis temperatures (850 °C and above), Raman spectroscopy identified two distinct bands at 484 cm^–1^ and 590–595 cm^–1^, unequivocally confirming the formation of the spinel LT-LCO phase alongside the desired layered LCO structure (595 cm^–1^ corresponding to XRD ICSD code 182346).

TGA showed thermal decomposition behaviors of particles synthesized from nitrate and acetate precursors (Figure 3e,f, respectively with the precursor metal sources in Figure S3). LiNO_3_ was found to decompose into Li_2_O at approximately 700 °C, while Co(NO_3_)_2_·6H_2_O transitions to its oxide form above 300 °C, in agreement with documented thermal decomposition pathways.^37^

Significantly, the TGA profiles for nitrate-derived particles reveal two distinct weight loss stages: the initial stage, occurring below 200 °C, primarily due to the evaporation of water, and the second stage around 450 °C, attributed to the decomposition of nitrate to oxide. This latter stage shows a marked reduction in weight loss from approximately 33% at 600 °C to about 7% at 1000 °C, indicating a substantial conversion of the particles to their oxide forms with increasing synthesis temperature.

Similarly, particles derived from acetate exhibited two primary weight loss regions below 200 °C, attributed to absorbed and lattice water loss. The second region, occurring around 450 °C, was a result of the decomposition of the metal acetate into oxide.^20^Figure S3 illustrates the decomposition of the metal acetate precursors, namely, LiC_2_H_3_O_2_·2H_2_O and Co(C_2_H_3_O_2_)_2_·4H_2_O, into oxides, occurring above 460 and 360 °C, respectively. Additionally, the weight loss due to acetate decomposition decreased at higher synthesis temperatures, from 37% at 600 °C to around 5% at 1000 °C, indicating that higher synthesis temperatures promote oxide formation.

**Figure 4:**
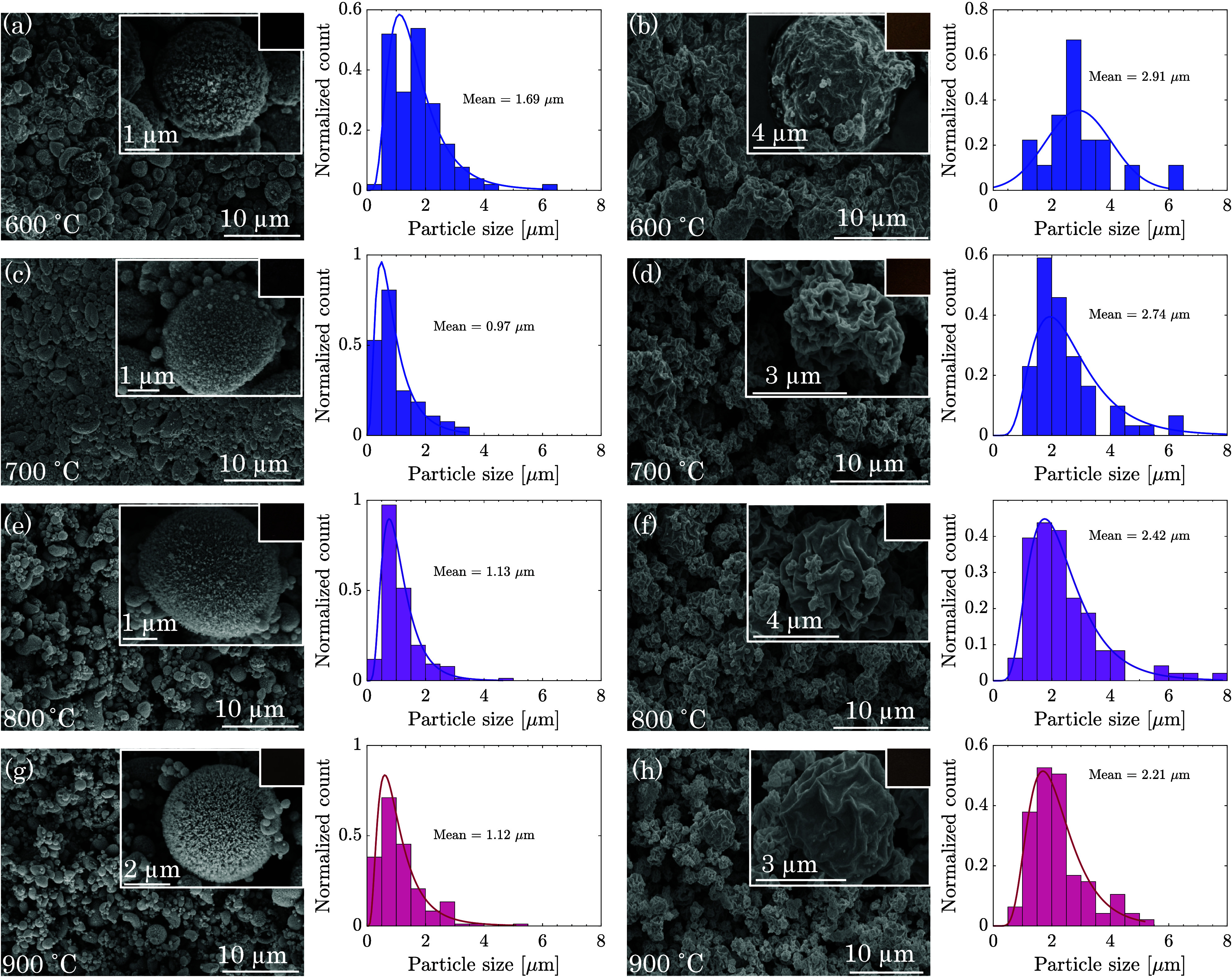
SEM images, including both lower and higher magnifications, along with the corresponding size distribution data and color variations at different temperatures for particles synthesized from nitrate precursors (a, c, e, g) and acetate precursors (b, d, f, h) at increasing reactor temperatures 600–900 °C. Particles derived from nitrate precursors exhibit a spherical morphology, while acetate precursors powder irregular shapes.

The morphological characteristics of the as-synthesized particles were elucidated using SEM, as demonstrated in Figures 4 and S4. Both nitrate and acetate precursors yielded hollow or shell-like particles, with TEM (Figure S5) further revealing the distinct morphologies. Particles from nitrate precursors displayed spherical structures, whereas acetate-derived particles exhibited irregular shapes, suggesting different mechanisms of formation influenced by the solvent evaporation rate to solute diffusion ratio within droplets. Rapid evaporation at the droplet’s periphery, particularly for solutes with low melting points, leads to the formation of shell-like structures.^32^ Nitrates promote better surface permeability, leading to the formation of hollow spheres with a thick crust. Conversely, acetate metals develop thin, poorly permeable crusts that impede the release of volatile species during decomposition. The increase in gas pressure inside the thin crust at elevated temperatures induces shell expansion, while subsequent pressure reduction during quenching and collection leads to shell collapse, resulting in a wrinkled surface.^38,39^

At synthesis temperatures of 600 and 700 °C, nitrate-derived particles showed aggregation and hygroscopic properties due to the presence of undecomposed metal. However, as the synthesis temperature increased, this aggregation decreased, and particles evolved into hollow spherical or ellipsoidal shapes with nanometer-sized primary particles on their surface, as evidenced in Figure S5. SEM analysis, quantified using ImageJ software, indicated particle diameters ranging from 0.8 to 1.7 μm, with size reduction observed at elevated temperatures (950 and 1000 °C), likely due to enhanced oxide conversion as supported by TGA findings. Acetate-derived particles, on the other hand, demonstrated aggregation at lower temperatures (600 °C) with an average size of about 3 μm, which decreased to approximately 1 μm at 1000 °C, aligning with observations of particle size reduction at increased reactor temperatures in prior studies.^22^ The color variation from dark to light brown for nitrate-derived particles and the reverse for acetate-derived particles with increasing temperature further reflects the influence of grain size and oxide type on the particles’ optical properties (see insets of Figures 4 and S4).

### Structural Evolution of LCO

**Figure 5:**
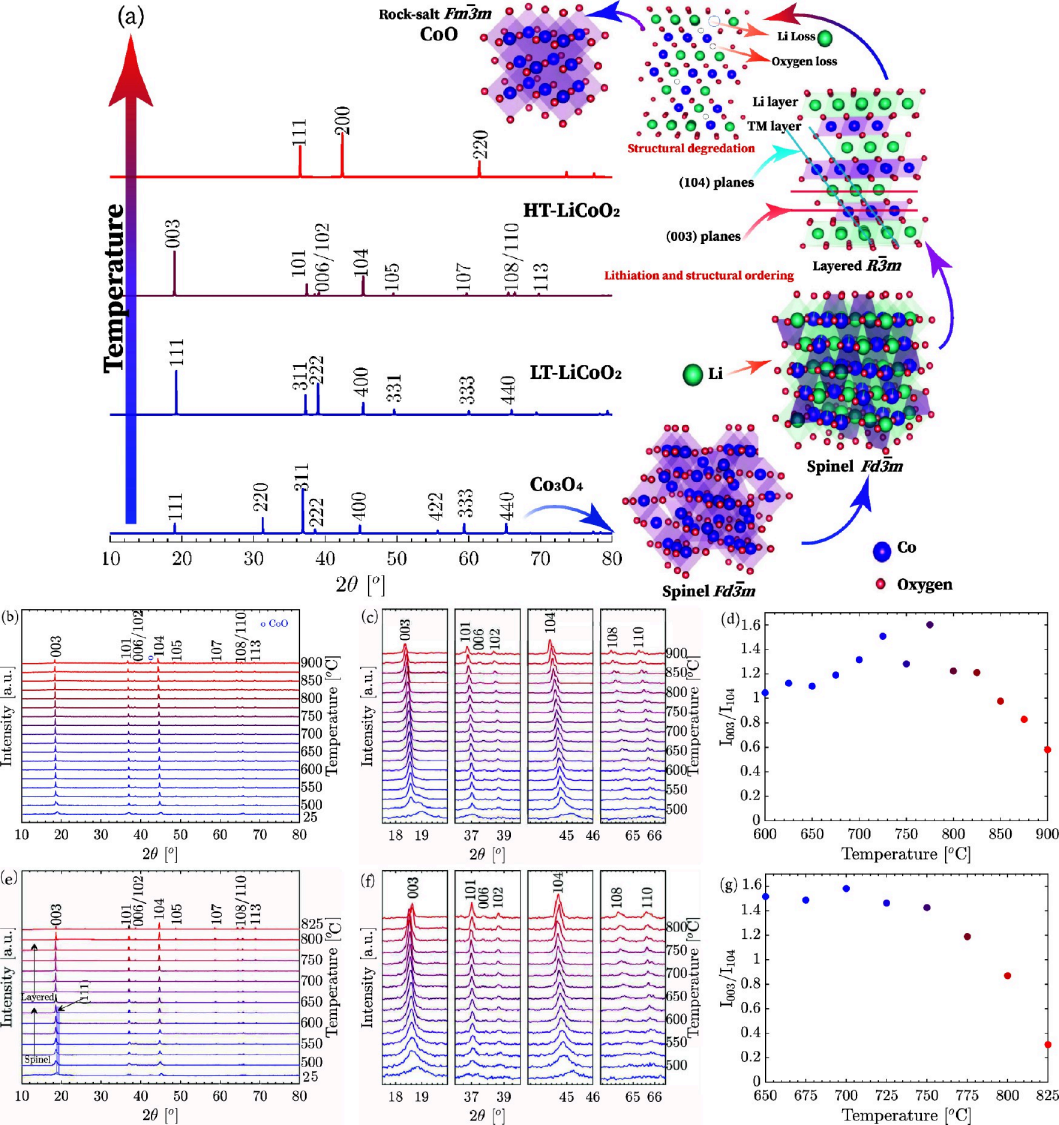
(a) Schematic representation of the structural evolution and phase transitions observed during the heating of synthesized particles. This sequence highlights the progression from the initial Co_3_O_4_ phase (ICSD 24210) to Li_2_Co_2_O_4_ (LT-LCO, ICSD 74320), and ultimately to the layered oxide structure LiCoO_2_ (HT-LCO, ICSD 51182). Structural defects become evident as LiCoO_2_ decomposes into CoO (ICSD 9865) due to the loss of lithium and oxygen at elevated temperatures. HT-XRD analysis is detailed for (b–d) particles derived from nitrate precursors at 800 °C, showing crystal patterns, a selected 2θ range, and the peak intensity ratio of (003) to (104), indicating phase purity and structural integrity. (e–g) The analysis extends to particles synthesized from acetate precursors at 900 °C, emphasizing the different structural outcomes based on precursor composition and thermal treatment.

To determine the structural evolution of spray-pyrolyzed particles from different metal salts across a spectrum of reactor temperatures, HT-XRD analysis was performed (Figure 5).

HT-XRD analysis at 800 °C for particles derived from metal nitrate (Figure 5b,c), corroborated by Raman spectroscopy, confirmed the coexistence of Co_3_O_4_ and spinel LT-LCO phases. Notably, the XRD profile displayed broad peaks of lower intensity due to the brief scanning duration of 10 min, in contrast to the more defined peaks obtained from powder X-ray diffraction (PXRD) with longer scanning times. At room temperature, the XRD peaks indicated limited crystallinity, aligning with previously reported studies.^40^

Elevation to 500 °C revealed the (003) peak of HT-LCO at an 18.6° 2θ position, indicative of the copresence of layered and spinel LCO phases up to 575 °C. Subsequent heating to 600 °C resulted in the disappearance of the spinel phase’s (111) peak, with a pure layered LCO phase emerging and remaining stable up to an annealing temperature of 850 °C. Beyond 875 and 900 °C, structural defects were observed as LCO decomposed into CoO, attributable to Li_2_O and O_2_ loss,^41^ potentially impacting electrochemical performance.

The ratio of integrated intensities *I*_003_/*I*_104_ serves as an indicator of cation mixing within the structure, while the splitting between the reflections of (006)/(102) and (108)/(110) provides insight into the well-defined nature of the layered oxide structure, as noted in the work by Wicker et al.^42^ In the context of the layered oxide structure displayed in Figure 5a, lithium ions are typically situated in the interlayer regions between the metal oxide layers, aligning with the positions of the (003) planes. Consequently, when cobalt migrates to the lithium sites, it leads to a reduction in the intensity of the (003) peak while leaving the (104) diffraction peak unaffected. Hence, a lower *I*_003_/*I*_104_ ratio, typically below 1.2, indicates a greater degree of cation mixing. Within the temperature range spanning 700 to 800 °C, an *I*_003_/*I*_104_ ratio exceeding 1.2 suggests a well-ordered cation arrangement within the structure. However, at higher temperatures, this ratio decreased due to the loss of Li_2_O, as evidenced by the reduced intensity of the (003) peaks, as seen in Figure 5d. Raising the synthesis temperature to 900 °C resulted in a narrower range of annealing temperatures that promote the formation of a layered oxide structure (Figure S6a–c).

Particles synthesized from acetate precursors at lower temperatures of 700 and 900 °C are illustrated in Figures 5 and S6, respectively. At 700 °C, the particles formed a spinel phase at lower temperatures and showed the coexistence of a layered LCO phase at higher temperatures (Figure S6d–f). However, at the higher synthesis temperature of 900 °C, distinct phases were observed. These included the spinel LT-LCO phase, represented by the (111) peak, which was observed up to 625 °C. Additionally, a layered HT-LCO phase with clear (006)/(102) and (108)/(110) reflections was observed, splitting up to 775 °C. It is evident that due to the lower decomposition of [C_2_H_3_LiO_2_·2H_2_O] compared to LiNO_3_, as shown in Figure S3, and the lower melting point of 58 °C for lithium acetate compared to 253 °C for lithium nitrate,^31^ the synthesis of HT-LCO is feasible at lower annealing temperatures using acetate precursors.^43^ This is supported by the high *I*_003_/*I*_104_ values observed at lower HT-XRD temperatures (Figure 5g).

### Effect of Annealing Conditions

**Figure 6:**
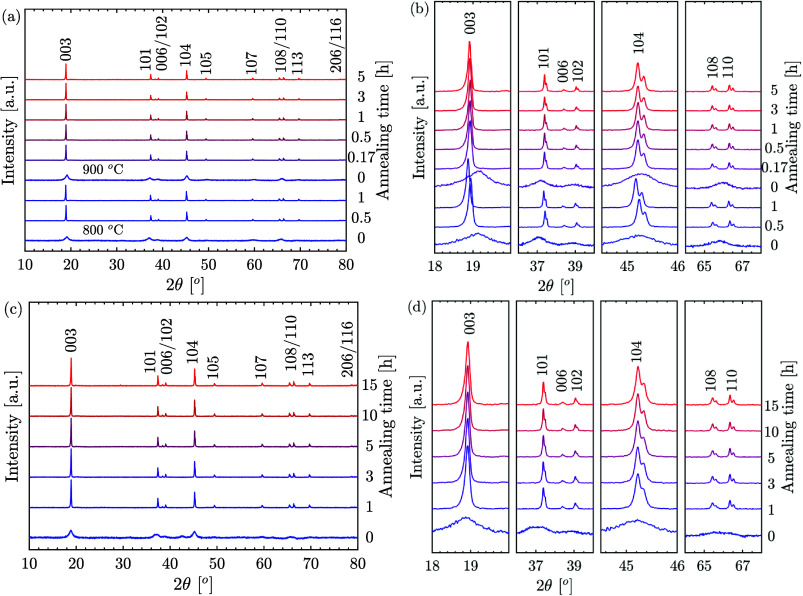
XRD patterns and selected 2θ ranges of the annealed LCO cathode materials. (a, b) XRD results for the materials synthesized from nitrates at reactor temperatures of 800 and 900 °C, followed by annealing at 775 °C. (c, d) XRD patterns of the materials prepared from acetate precursors synthesized at 900 °C and annealed at 750 °C for varying annealing times.

The optimal annealing temperatures for synthesizing LCO particles with a layered structure were determined through HT-XRD analysis (refer to Figure 5). For particles obtained from nitrate precursors at 800 and 900 °C, a high *I*_003_/*I*_104_ ratio and distinct separation of (006)/(102) and (108)/(110) reflections were observed at 775 °C, indicating the formation of a well-layered structure. Therefore, a sintering temperature of 775 °C was selected for different annealing durations. On the other hand, particles synthesized from acetate precursors at 900 °C showed superior cation ordering at lower HT-XRD temperatures. Hence, an annealing temperature of 750 °C was chosen. Figure 6 presents the XRD patterns of the as-synthesized particles (0 h of annealing) and particles annealed for various durations.

For particles synthesized from nitrate precursors at 800 °C and annealed for 0.5 and 1 h, the XRD patterns showed all peaks indexed to the rhombohedral α-NaFeO_2_ structure with a space group of *R*3̅*m*. The high *I*_003_/*I*_104_ ratio (>1.2) and distinct separation of (006)/(102) and (108)/(110) peaks confirmed the formation of a well-layered structure with minimal cation mixing, as illustrated in Figure 6a,b, and detailed in Table 1. Rhombohedral layered oxide cathodes with an *I*_003_/*I*_104_ ratio below 1.2 were found to result in reduced reversible capacity, and ratios below 1 were electrochemically inactive.^42^ As shown in Figure 6a,b, a slight leftward shift observed in the 1 h annealing pattern was attributed to lattice expansion, supported by an increase in crystallite size from approximately 140 to 190 nm, calculated using the Williamson–Hall strain model. Similarly, particles synthesized at 900 °C exhibited a pure HT-LCO layered structure with good cation ordering, as evidenced by the high *I*_003_/*I*_104_ ratio observed across a range of annealing times from 10 min to 5 h, with the highest value obtained at 3 h of annealing.

**Table 1:** Chemical Composition and XRD Analysis of LiCoO_2_ Cathode Materials Synthesized from Nitrate and Acetate Precursors Using Different Synthesis and Annealing Conditions

precursor	synthesis temperature (°C)	annealing temperature (°C)	annealing time (h)	Li:Co	*I*_003_/*I*_104_	*x*	*c*/*a*	*R*_*wp*_ (%)	GOF
nitrate	800	775	0.5	1.04	1.42	1.00	4.9900	1.74	1.49
			1	1.00	1.30	1.00	4.9913	1.78	1.51
	900		0.17	1.08	1.26	1.00	4.9890	1.66	1.44
			0.5	1.03	1.28	1.01	4.9900	1.72	1.47
			1	1.05	1.36	0.99	4.9901	1.86	1.59
			3	1.04	1.61	1.00	4.9906	1.98	1.72
			5	1.05	1.34	1.01	4.9912	1.85	1.76
acetate	900	750	1	0.96	1.39	1.00	4.9916	1.67	1.45
			3	0.97	1.38	0.99	4.9915	1.64	1.42
			5	0.94	1.38	0.99	4.9913	1.64	1.43
			10	0.95	1.39	0.99	4.9915	1.70	1.47
			15	0.96	1.29	0.97	4.9914	1.65	1.42

The molar ratio between Li and Co, determined by MP-AES, closely approached stoichiometry for all the particles prepared from nitrate precursors and synthesized at different reactor temperatures, as shown in Table 1. Although a 5% excess of Li was used, some ratios were higher, which was also reported in other work^44^ and could be attributed to instrument uncertainty. The *R* factor is very sensitive to stoichiometry and serves as an additional indicator of the degree of ordering in layered oxide cathode materials. It is calculated as *R* = (*I*_006_ + *I*_102_)/*I*_101_ and is inversely proportional to the cation ordering in the structure.^45,46^ This factor can be used to evaluate the stoichiometry of the layered cathode oxides,^42^ where *R* = 4/3[(1.6 – *x*)/*x*]^2^ is used to evaluate *x* for Li_*x*_Co_2–*x*_O_2_.^42,46^ The results in Table 1 reveal *x* values indicative of materials closely approaching stoichiometry, and the chemical compositions of the cathode materials are nominal. Another measure for assessing cation ordering is the *c*/*a* ratio of cell parameters, where a larger value signifies better cation ordering.^46^ The refinement results, (using GSAS software), presented in Table 1, align with values reported in existing literature.^46^ The weighted profile (*R*_*wp*_) and goodness of fit (GOF) further affirm the accuracy of the refinement process.

For particles synthesized from acetate precursors were annealed at a marginally lower temperature, necessitating a longer annealing time to ensure complete conversion to the HT-LCO structure. The XRD patterns verified pure crystal formation without the presence of LT-LCO phases alongside the high *I*_003_/*I*_104_ ratio (>1.2) for all the samples and the refinement results. Although excess Li was employed in the preparation of acetate-derived particles, MP-AES measurements revealed slightly lower Li content compared to stoichiometry. This observation was also reflected in the *x* value calculated from the analytic approximation, particularly at the extended annealing time of 15 h. This discrepancy can be attributed to Li defects resulting from the lower decomposition temperature of Li acetate and Li loss at longer annealing times.

SEM images of the as-synthesized particles and the annealed particles of LCO from nitrate and acetate precursors are provided in Figures S7 and S8. For the particles synthesized from nitrate precursors and annealed for various durations, the initial hollow spherical morphology largely transformed into single-crystal particles. Over time, the primary nanometer-sized particles on the secondary particles grew into larger particles. Conversely, particles prepared from acetate precursors maintained their initial hollow, wrinkled surface morphology for up to 5 h of annealing time. However, these particles exhibited smaller primary particle sizes compared to those from nitrate precursors, displaying a more porous and agglomerated structure.

Typically, when primary particles are reduced in size, they tend to offer a greater specific surface area. This heightened surface area improves the electrolyte’s ability to wet the particles. Moreover, smaller particles lead to shorter diffusion distances for Li^+^ ions because the migration time of Li^+^ (*t*_Li^+^_), is linked to the diffusion length (*L*) and diffusion coefficient (*D*_Li^+^_) through the equation . However, it is worth noting that smaller particles also come with a drawback: They create more grain boundaries within the material. These grain boundaries can hinder electron transfer, leading to increased polarization. Conversely, larger particles have the potential to enhance tap density, which, in turn, can boost volumetric specific capacity. Nevertheless, larger particles face the challenge of microcracking during the intercalation and deintercalation processes of Li^+^ ions. This microcracking can have a detrimental effect on the cycle life of the battery.^47^ Therefore, achieving optimal battery performance involves synthesizing pure cathode materials with carefully selected particle sizes.

**Figure 7:**
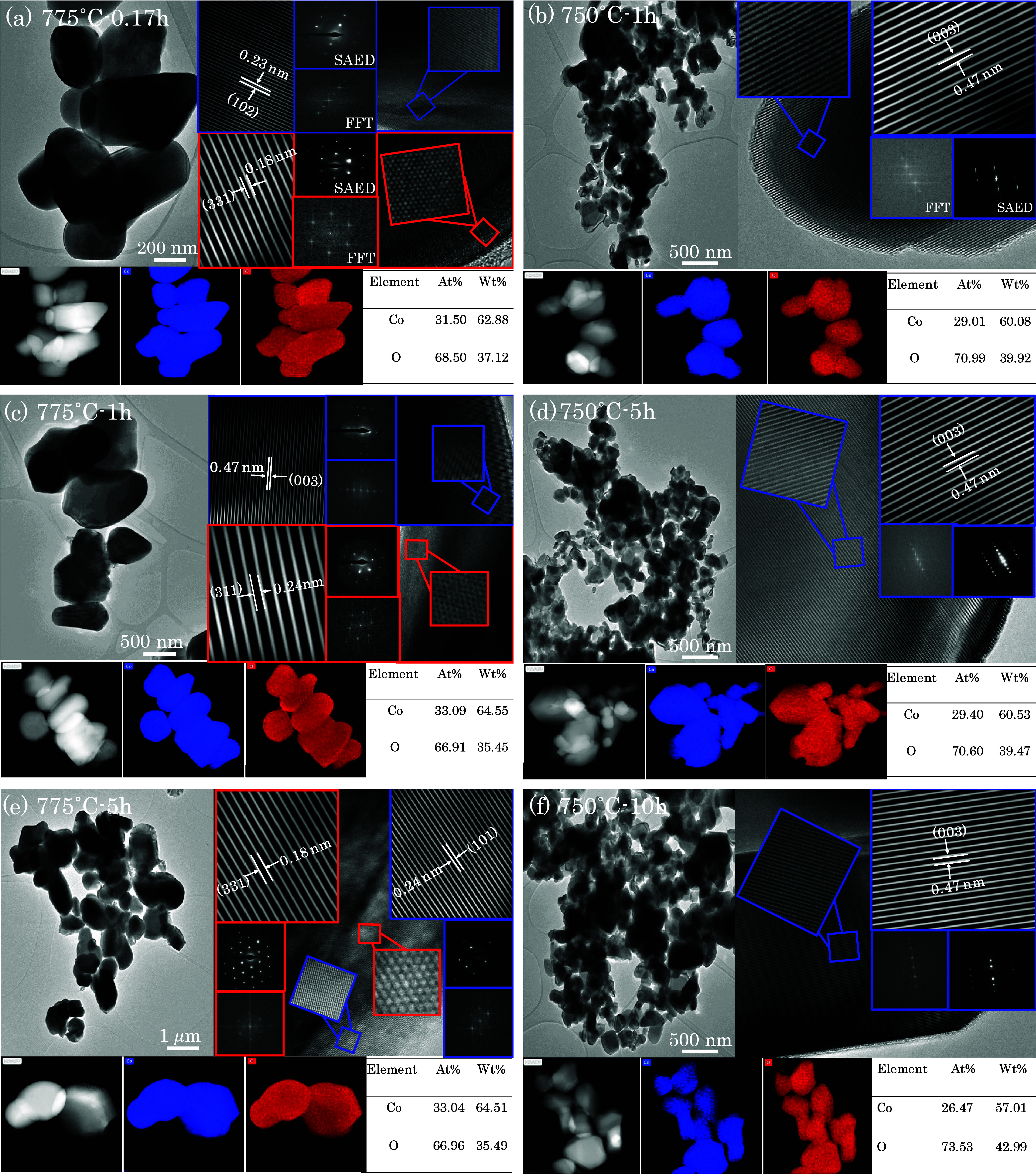
TEM images and corresponding EDS elemental maps, HRTEM, FFT, and SAED patterns of the annealed LCO particles. LCO particles were synthesized from nitrate precursors at 900 °C and annealed at 775 °C for (a) 0.17 h, (c) 1 h, and (e) 5 h, or from acetate precursors at 900 °C and annealed at 750 °C for (b) 1 h, (d) 5 h, and (f) 10 h.

The TEM results of the particles from nitrate precursors in Figure 7a,c,e, annealed at 775 °C for different durations, revealed mixed phases of spinel Co_3_O_4_ and the layered HT-LCO structure. Although XRD indicated a pure HT-LCO phase, TEM showed a mixed phase. The presence of the spinel phase is likely due to unreacted Co_3_O_4_, resulting from the high decomposition temperature of LiNO_3_ to Li_2_O at approximately 700 °C, compared to LiC_2_H_3_O_2_·2H_2_O at 460 °C. The lower decomposition temperature of lithium acetate resulted in pure HT-LCO at various selected annealing times. The SAED patterns aligned with existing literature,^48^ supporting the assertion that the use of acetate leads to the formation of HT-LCO at lower temperatures, as also confirmed by Carewska et al.^49^

**Figure 8:**
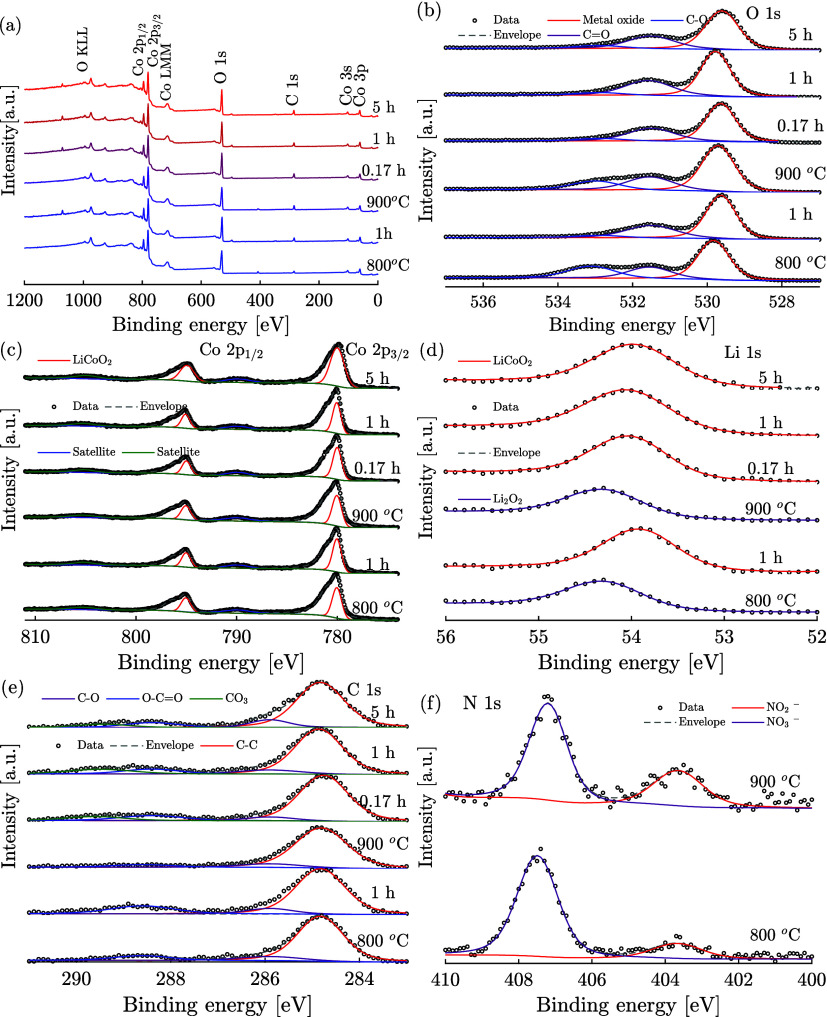
X-ray photoelectron spectroscopy (XPS) analysis of LCO cathode materials. The figure displays (a) XPS survey scans, as well as spectra for (b) O 1s, (c) Co 2p, (d) Li 1s, (e) C 1s, and (f) N 1s, for LCO cathode materials synthesized using nitrate precursor. Additionally, it includes data for two different synthesis temperatures and the annealed particles synthesized at the higher temperature.

XPS analysis was conducted to elucidate the surface chemical states of LCO particles, focusing on samples synthesized from nitrate precursors at 800 °C and subsequently annealed at 775 °C for one hour, alongside those prepared at 900 °C and subjected to various annealing periods. Detailed survey spectra and high-resolution scans for O 1s, Co 2p, Li 1s, C 1s, and N 1s core levels are depicted in Figure 8, providing comprehensive insights into the surface chemistry of the annealed LCO particles.

The O 1s spectra revealed peaks at approximately 529 eV, characteristic of lattice oxygen within the LiCoO_2_ structure, indicative of robust metal–oxygen (M–O) bonds. This observation is consistent with the interface studies of lithium cobalt oxide by Ferber et al.^50^ Peaks attributed to carbon–oxygen (C–O) bonds and carbonyl (C=O) functionalities suggest the presence of surface carbonates and organic residues, aligning with prior findings by Haasch et al.^51^ Notably, the C 1s spectra exhibit a prominent peak for C–C bonding around 285 eV, hinting at hydrocarbon contamination. Additionally, despite the absence of Li_2_CO_3_ in the XRD patterns, the detection of carbonate groups in the C 1s spectra for samples processed at 900 °C and annealed over varied durations underscores the likelihood of surface carbonate formation, possibly through LiCoO_2_–CO_2_ interactions leading to Li_2_CO_3_ synthesis.

Analysis of Co and Li core levels revealed Co 2p_3/2_ and 2p_1/2_ peaks at 780 and 795 eV, respectively, affirming the LiCoO_2_ phase presence. Shoulders adjacent to these peaks suggest minor Co_3_O_4_ incorporation within the LCO matrix, corroborated by Cole et al.^52^ The Li 1s peak situated at 45 eV, characteristic of annealed samples, directly corresponds to LiCoO_2_, while a slight peak shift observed in as-synthesized samples implies Li_2_O_2_ presence, harmonizing with complementary XRD and Raman analyses which emphasize the transformative impact of annealing toward achieving optimal LiCoO_2_ structuring.

Furthermore, N 1s spectra disclosed signatures of nitrates and nitrites within the as-synthesized samples, with a notable intensity augmentation in samples synthesized at elevated temperatures, suggesting a temperature-facilitated nitrate to nitrite conversion. This aligns with thermal gravimetric analysis results, which indicate a more efficient oxide conversion at raised synthesis temperatures. Annealing effectively eradicates nitrate and nitrite traces, confirming their complete decomposition to oxides and highlighting the critical role of thermal processing in optimizing the chemical purity and structural integrity of LCO cathodes.

XPS was also employed to study the surface chemical compositions of the particles derived from acetate precursor, processed at 900 °C and subjected to annealing at 750 °C for varied time spans. A comparative analysis was also conducted for samples synthesized at a lower benchmark of 800 °C, as depicted in Figure S9.

A distinct characteristic of acetate-derived particles was the pronounced carbonate signature, underscored by CO_3_ peaks within the C 1s spectra, indicative of a heightened carbonate concentration. This feature was particularly prominent in as-synthesized particles, suggesting an incomplete oxide conversion during the synthesis phase. The Li 1s spectra further corroborated this observation, revealing Li_2_CO_3_ presence in the as-prepared samples, thereby marking a notable deviation in surface chemistry attributable to precursor selection.

### Electrochemical Performance

**Figure 9:**
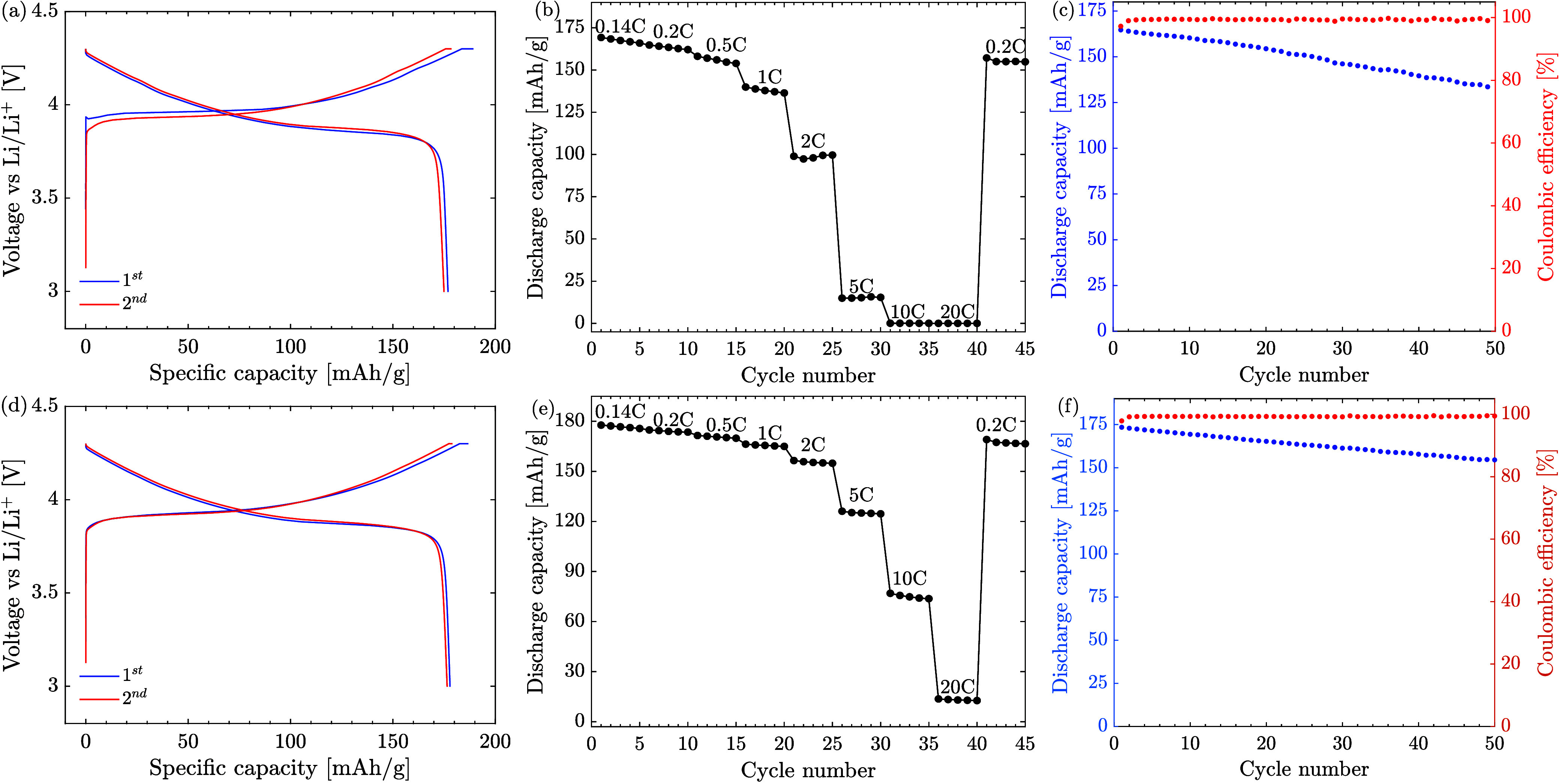
Electrochemical performance assessment of LCO particles synthesized from nitrate precursors at 800 °C and annealed at 775 °C for (a–c) 30 min and (d–f) 1 h. (a,d) Initial charge and discharge curves at 0.1*C*, (b,e) rate performance and (c,f) cycling performance. The experiments were conducted using a 90:5:5 ratio of active material:carbon:binder and 20 μL of electrolyte.

We assessed the effectiveness of the selected annealing conditions in synthesizing LCO particles for battery cathodes through electrochemical tests using half-cells with a Li-metal counter electrode. The LCO particles, synthesized from nitrate precursors at 800 °C and annealed at 775 °C for durations of 30 min and 1 h, exhibited promising results, as depicted in Figure 9. Particles annealed for 30 min showcased an initial discharge capacity of 176.8 mAh/g with an initial Coulombic efficiency (CE) of 93.5%. Remarkably, particles annealed for 1 h exhibited a slightly higher capacity of 177.9 mAh/g with an initial CE of 95%. These results surpassed previous literature values under similar synthesis methods, voltage window, and 0.1*C* rate,^9,12−14^ where reported capacities typically remained around 150 mAh/g or lower.

Cycling performance at 0.5*C* revealed promising capacity retention, with the 1 h annealed particles retaining 89% after 50 cycles and 82% capacity after 115 cycles (Figure S10), compared to 81% for the 30 min annealed particles after 50 cycles. Moreover, rate capability tests demonstrated excellent capacity retention even under high cycling rates. For instance, the 30 min annealed particles maintained an average capacity retention of 58% at 5*C*, improving to 91% after 45 cycles at 0.2*C*. Conversely, the 1 h annealed particles exhibited better performance, with an average capacity retention of 70% at 5*C*, 42% at 10*C*, and 93% at 0.2*C* after 45 cycles.

Conversely, particles synthesized from acetate precursors at 900 °C and annealed at 750 °C underwent testing with a ratio of active material, carbon, and binder of 80:10:10 and 80 μL of electrolyte to optimize the electrolyte’s wettability across porous particle aggregates. Notably, these acetate-derived particles, particularly those annealed for 3 and 15 h, exhibited initial discharge capacities at 0.1*C* of 135 mAh/g and 136.4 mAh/g, respectively, with commendable capacity retention of approximately 79% after 50 cycles at 0.5*C*, as shown in Figure S11.

Achieving optimal battery performance thus requires multiparameter optimization to synthesizing pure layered oxide cathode materials, tailoring particle sizing, and refining electrode fabrication techniques.

## Conclusions

This study provides significant insights into the synthesis pathway of layered-oxide LCO cathode materials via spray pyrolysis. By systematically investigating the effect of precursor choice and annealing temperature on the morphology, structure, and electrochemical performance of LCO particles, we have uncovered several key aspects that advance the understanding of high-performance cathode synthesis.

Our findings reveal distinct differences in morphology and structure of LCO particles synthesized from nitrate and acetate precursors. Nitrate-derived particles exhibit hollow, spherical shapes, while acetate-derived particles display irregular morphologies. This disparity arises from differences in precursor decomposition behavior and resulting kinetics during spray pyrolysis. Nitrate-derived LCO particles at lower temperatures exhibit phase instability due to undecomposed nitrate, transitioning to Co_3_O_4_ spinel phase with increased synthesis temperature, potentially forming partially lithiated Co_3_O_4_. Conversely, acetate-derived particles show absence of undecomposed precursors at lower temperatures, forming rock-salt CoO initially and transitioning to Co_3_O_4_ as the dominant phase at higher temperatures, confirmed by Raman spectroscopy.

Through the application of high-temperature XRD, we have clarified the structural evolution of these particles, identifying the formation of layered and spinel LCO phases at different annealing temperatures for each precursor type. By selecting optimum annealing conditions based on HT-XRD results, we demonstrate the synthesis of well-layered LCO structures with excellent electrochemical performance. Nitrate-derived particles annealed at 775 °C exhibit initial discharge capacities surpassing literature values, while acetate-derived particles annealed at 750 °C show capacity retention of approximately 79% after 50 cycles.

These insights into the optimization of LCO cathode materials not only deepen our understanding of the synthesis process but also pave the way for the development of high-performance batteries with improved efficiency, durability, and capacity. By clarifying the impact of precursor choice and annealing conditions on the structural and electrochemical properties of LCO, this study gives measurement principles for the design and synthesis of advanced cathode materials for next-generation lithium-ion batteries.

## Supporting Information Available

Additional figures and data, including: size distribution of precursor droplets; phase diagrams of lithium and cobalt oxides; thermal analysis of precursor materials; additional SEM/TEM images of as-synthesized particles; supplementary high-temperature XRD patterns and analysis; SEM images of annealed particles; XPS data for acetate-derived particles; extended cycling data; electrochemical testing results for acetate-derived particles; and details of material characterization techniques (PDF)
