# Supplementary material for: Synthesis Pathway of Layered-Oxide Cathode Materials for Lithium-Ion Batteries by Spray Pyrolysis
Source: ACS Appl Mater Interfaces. 2024 Jun 24;16(26):33633–46. doi: 10.1021/acsami.4c06503 (PMC11231976; doi:10.1021/acsami.4c06503)
Supplement: Supplementary file 1 — am4c06503_si_001.pdf [file am4c06503_si_001.pdf]

Supporting Information

Synthesis Pathway of Layered-Oxide Cathode Materials for Lithium-Ion Batteries by Spray Pyrolysis

Manar Almazrouei,[*,†,‡] Sulki Park,[†] Maurits Houck,[†,¶] Michael De Volder,[†] Simone Hochgreb,[†] and Adam Boies[*,†]

†*Department of Engineering, University of Cambridge, Cambridge CB2 1PZ, United Kingdom*

‡*Department of Mechanical and Aerospace Engineering, United Arab Emirates University, Al Ain 15551, Abu Dhabi, United Arab Emirates*

¶*Echion Technologies, Ltd., Sawston, Cambridge CB22 3FG, United Kingdom*

E-mail: manar.almazrouei@uaeu.ac.ae; amb233@cam.ac.uk

Material Characterization Techniques

Characterization of the synthesized LCO employed a suite of analytical techniques to elucidate crystal structure, elemental composition, and microstructural properties.

XRD was performed on PANalytical Empyrean and X'Pert PRO diffractometers, using CuKα radiation. Measurements covered a 2θ range from 10° to 80°, with a step size of 0.02°, spanning 30 to 60 minutes. The GSAS-II package facilitated data refinement, referencing the α-$NaFeO_2$ structure within the $R\bar{3}m$ space group. HT-XRD analysis utilized a PANalytical Empyrean instrument integrated with an Anton-Paar XRK 900 heating furnace. Samples, positioned on Macor ceramic holders, were examined under a consistent airflow of 0.5 L/min. A temperature ramp of 5 °C/min was applied, with each temperature point scanned for 10 minutes following a 30-minute stabilization period.

Raman Spectroscopy was conducted to study the molecular composition and vibrational properties of materials using a Horiba Explora Plus instrument equipped with a 532 nm laser. Spectra, recorded across a range of 50 to 3000 cm^{-1}, employed a laser power of 1%. Care was taken to minimize laser power to 1% to prevent phase degradation, a precaution stemming from observations that increased laser power can induce the transformation of rock-salt CoO to spinel Co_3O_4.[1]

Thermal Stability was assessed through thermogravimetric analysis (DSC/TGA) using a Discovery SDT 650 instrument. Samples were subjected to a heating protocol up to 1000 °C at a rate of 10 °C/min, under a constant airflow of 100 ml/min.

Elemental Analysis and lithium-to-metal ratio determinations were achieved using Microwave Plasma Atomic Emission Spectroscopy (MP-AES) on an Agilent 4210 instrument, providing precise compositional data.

SEM imaging was performed on a TESCAN MIRA3 FEG-SEM and a FEI Magellan 400 microscope, operating at 5 kV. Prior to imaging, samples were sputter-coated with a 10 nm platinum layer to enhance conductivity and image quality. Additionally, some images were captured using a FEI Magellan 400 microscope with back-scattered electron detectors.

TEM, Energy-Dispersive X-ray Spectroscopy (EDS), High-Resolution TEM (HRTEM), Selected-Area Electron Diffraction (SAED), and Fast Fourier Transform (FFT) analyses were integral for

examining the particles' detailed morphology, elemental distribution, and localized structure. Sample preparation entailed drop-casting a particle dispersion in isopropyl alcohol onto lacey carbon-coated 300 mesh copper grids. Analyses were conducted using a Thermo Scientific Talos F200X G2 TEM at 200 kV. TEM images were captured with a Ceta 16M CMOS camera. EDS mapping and spectra, performed in STEM mode using the HAADF detector, utilized the Super-X EDS system with four silicon drift detectors, enabling precise elemental analysis. HRTEM provided lattice fringe visualization and d-spacing measurements, facilitated by ImageJ's FFT function. SAED patterns offered additional crystallographic data, enriching the structural analysis.

X-Ray Photoelectron Spectroscopy (XPS) examinations were conducted on a ThermoFisher ESCALAB XI+ instrument to analyze surface chemistry and electronic states. Operating conditions were optimized for accuracy, with spectra acquisition occurring under a vacuum maintained above 5x10^{-1} mbar by an Edwards E2M28 pump. Argon was the primary background gas during sample handling. The Al Kα source, monochromated to 1486.68 eV, alongside software-calibrated work functions (4.6 eV), ensured precise energy resolution (0.1 eV for high-energy and 1 eV for survey spectra). Detailed lithium analysis involved 50 scans to enhance signal quality over an analysis area of 650 μm. Prior to analysis, samples were swiftly transferred to a spectrometer lock chamber under a vacuum of 2x10^{-6} mbar, facilitated by an Edwards RV5 pump. Charge compensation was applied using a flood gun (100 μA) to mitigate charging effects, crucial for reliable XPS data.

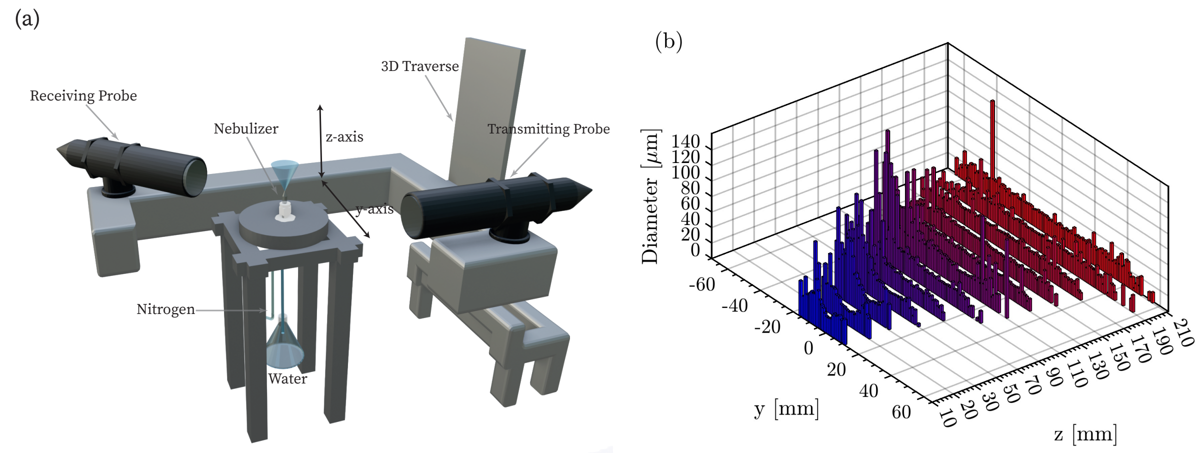

Figure S1: The experimental setup of the Phase Doppler Anemometry (PDA) system is shown in (a). The droplet diameters are illustrated in (b) with variation along the y-direction and different heights above the nebulizer in the z-direction.

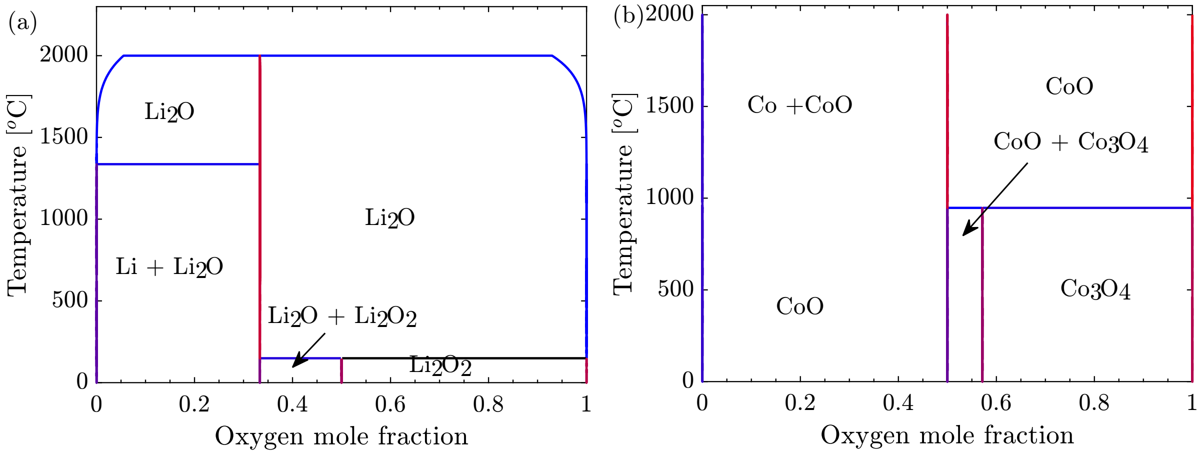

Figure S2: Phase diagrams of lithium (a) and cobalt (b), providing insights into the thermal stability and phase transitions relevant to LCO cathode material synthesis.

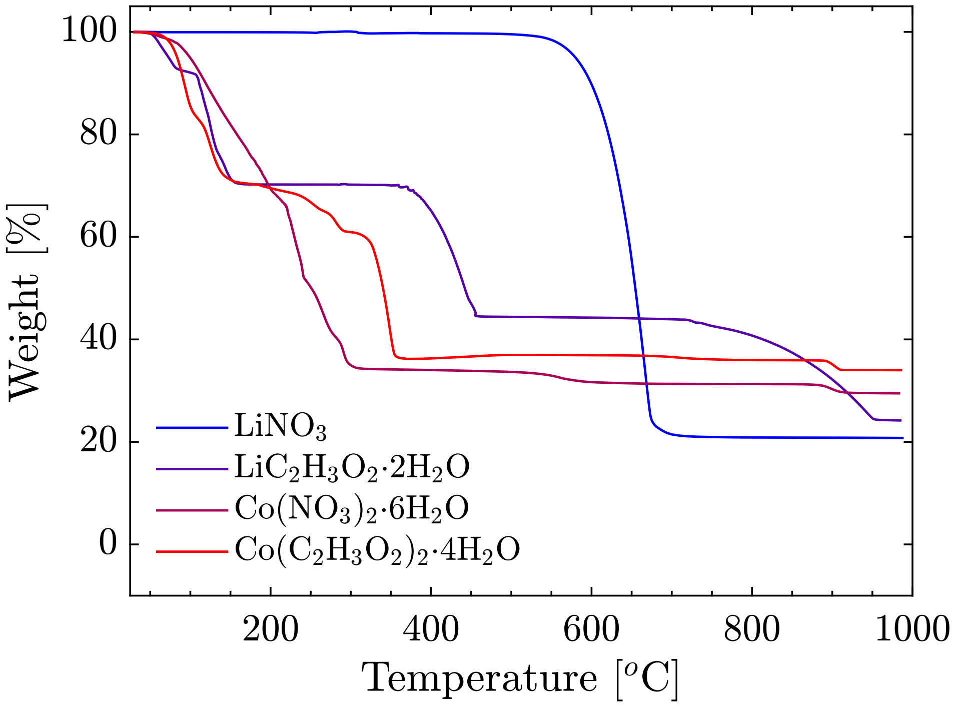

Figure S3: TGA of lithium and cobalt precursors, highlighting their decomposition profiles to guide the synthesis of layered oxide cathode materials.

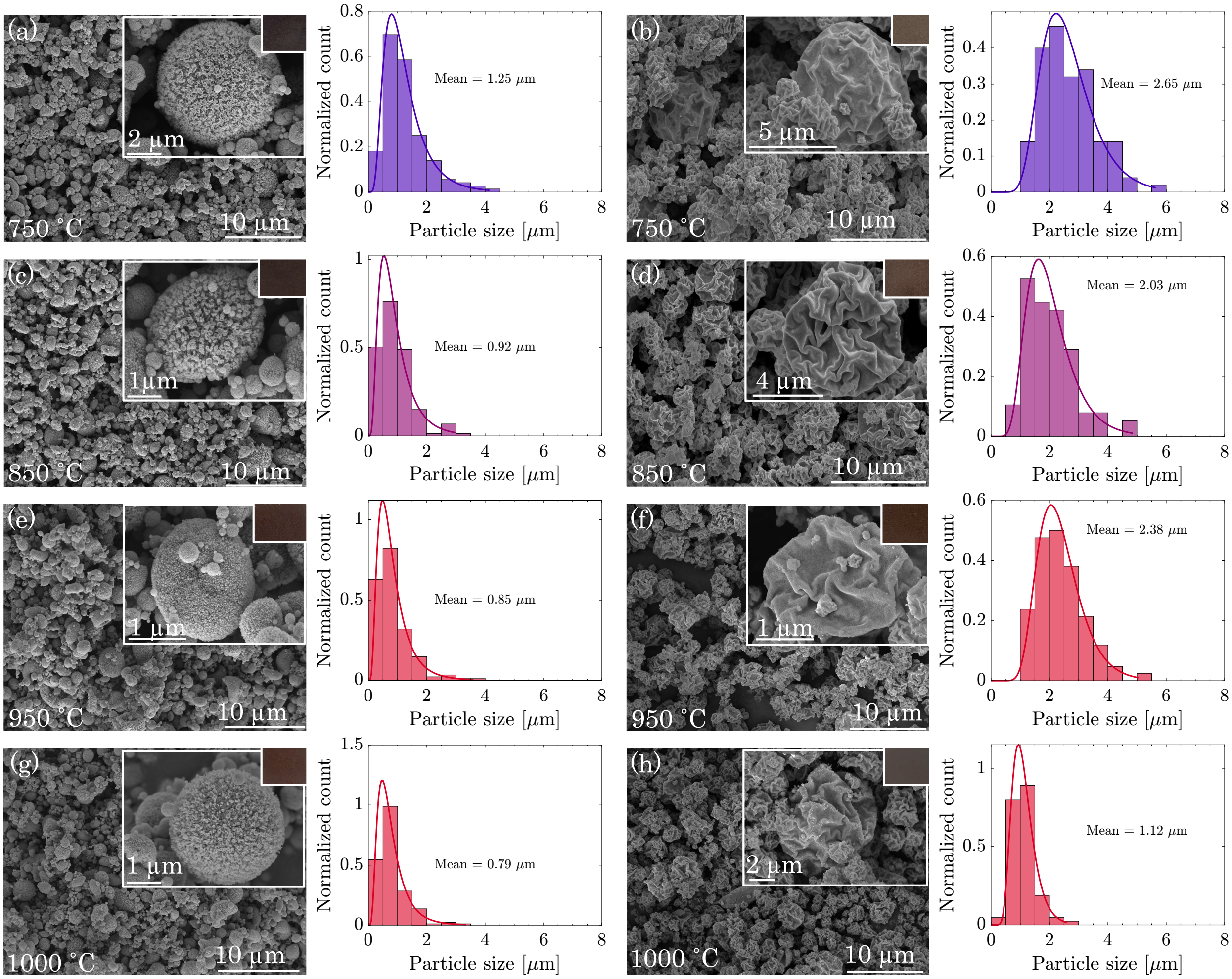

Figure S4: SEM images and the corresponding particle size distribution analysis at various synthesis temperatures for LCO particles derived from nitrate (a, c, e, g) and acetate (b, d, f, h) precursors, illustrating the temperature-dependent morphological evolution.

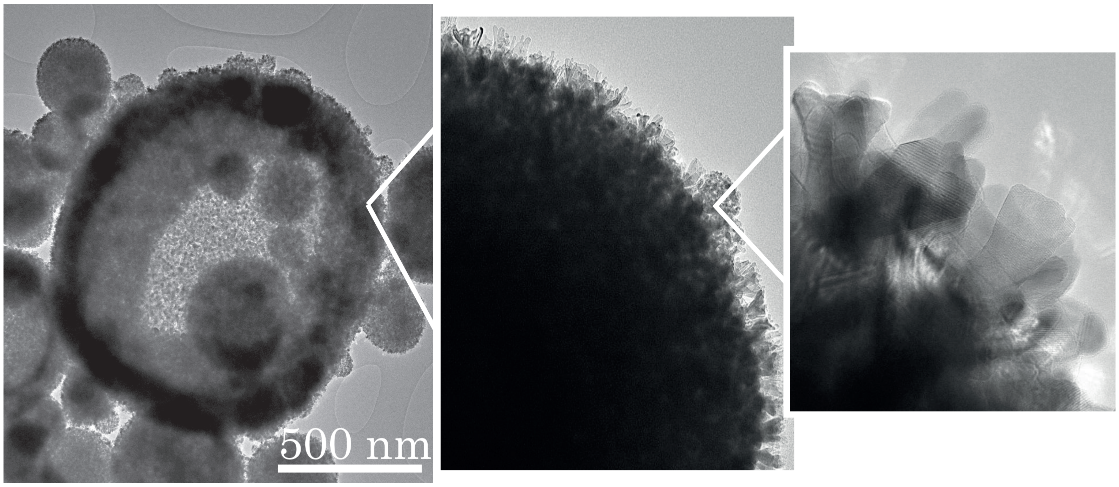

Figure S5: TEM images showcasing the hollow structural morphology of LCO particles synthesized from nitrate precursors at 900°C, emphasizing the influence of synthesis temperature on particle architecture.

Structural Evolution of LCO

While the HT-XRD temperature range of the particles synthesized from nitrate precursors at 900 °C as shown in Figures S6a-c was restricted to 600-900 °C to minimize Li defects at elevated temperatures, the presence of the spinel phase, indicated by the 111 peak, persisted until a high temperature of 725 °C. This observation can be explained by the TGA results (refer to Figure 3), where particles synthesized at higher temperatures exhibited a higher degree of thermal decomposition into the LT-LCO phase. Consequently, achieving a pure layered oxide structure necessitates higher annealing temperatures of the as-synthesized particles. Furthermore, it should be noted that an elevated reactor temperature can lead to an increased loss of Li and oxygen at higher annealing temperatures, as evidenced by the lower I_{003}/I_{104} ratio in Figure S6c when compared to particles synthesized at lower temperatures.

The HT-XRD results for the particles synthesized from acetate precursors at 700 °C are depicted in Figures S6d-f. Upon analysis, the presence of Co_3O_4 and CoO phases in the as-synthesized particles at room temperature (25 °C) was identified. With the temperature rising to 500 °C during the HT-XRD measurements, three distinct peaks emerged at 18.8°, 37.2°, and 44.99° 2θ. Notably, these peaks were observed at higher 2θ values compared to the peak positions of the layered HT-LCO structure's (003), (101), and (104) peaks,[2] suggesting the presence of the (111), (311), and (400) peaks of the spinel LT-LCO phase. A subtle splitting of the (006)/(102) and

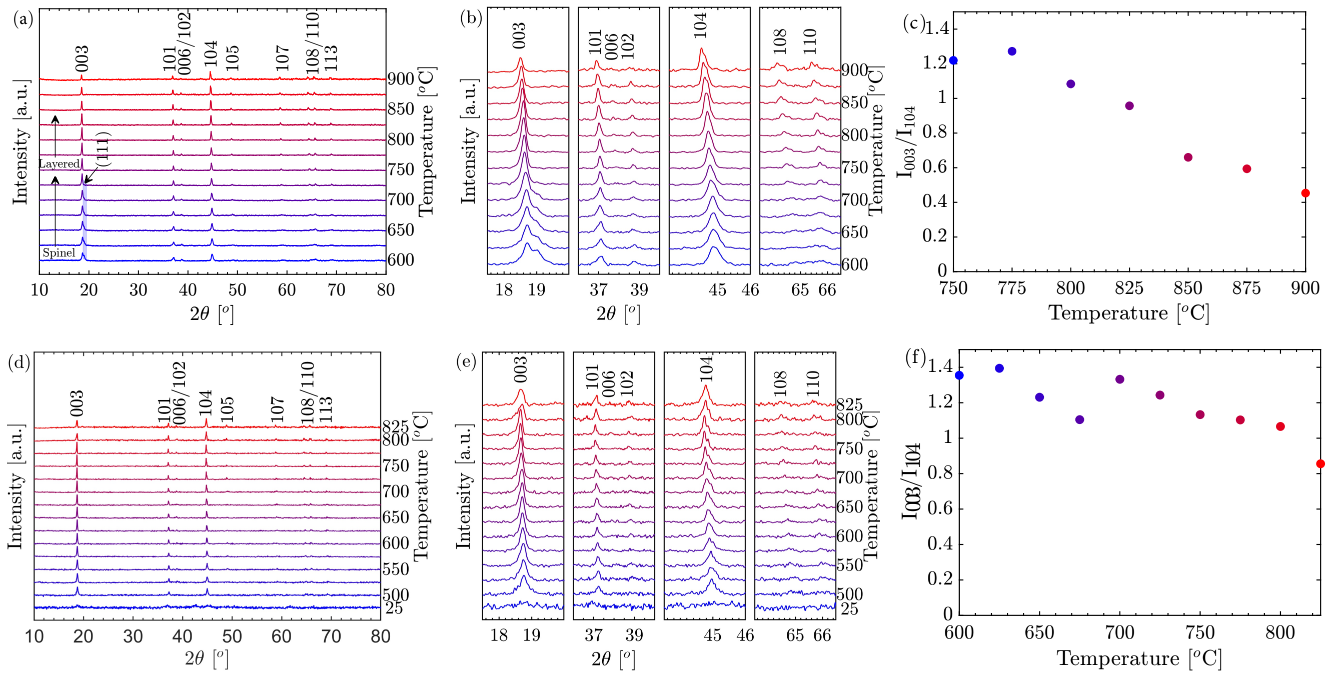

Figure S6: HT-XRD analysis of (a) crystal patterns, (b) selected 2θ range, and (c) peak intensity ratio of (003) and (104) for SP LCO particles synthesized from nitrate precursors at (a-c) 900 °C and LCO particles synthesized from acetate precursors at (d-f) 700 °C.

(108)/(110) reflections was discerned within the temperature range of 550 to 775 °C, implying the coexistence of a layered LCO phase alongside the spinel phase, as evident by the broad peaks. However, the emergence of Li defects was observed at a temperature of 800 °C, correlating with the decline in the I_{003}/I_{104} value at higher temperatures.

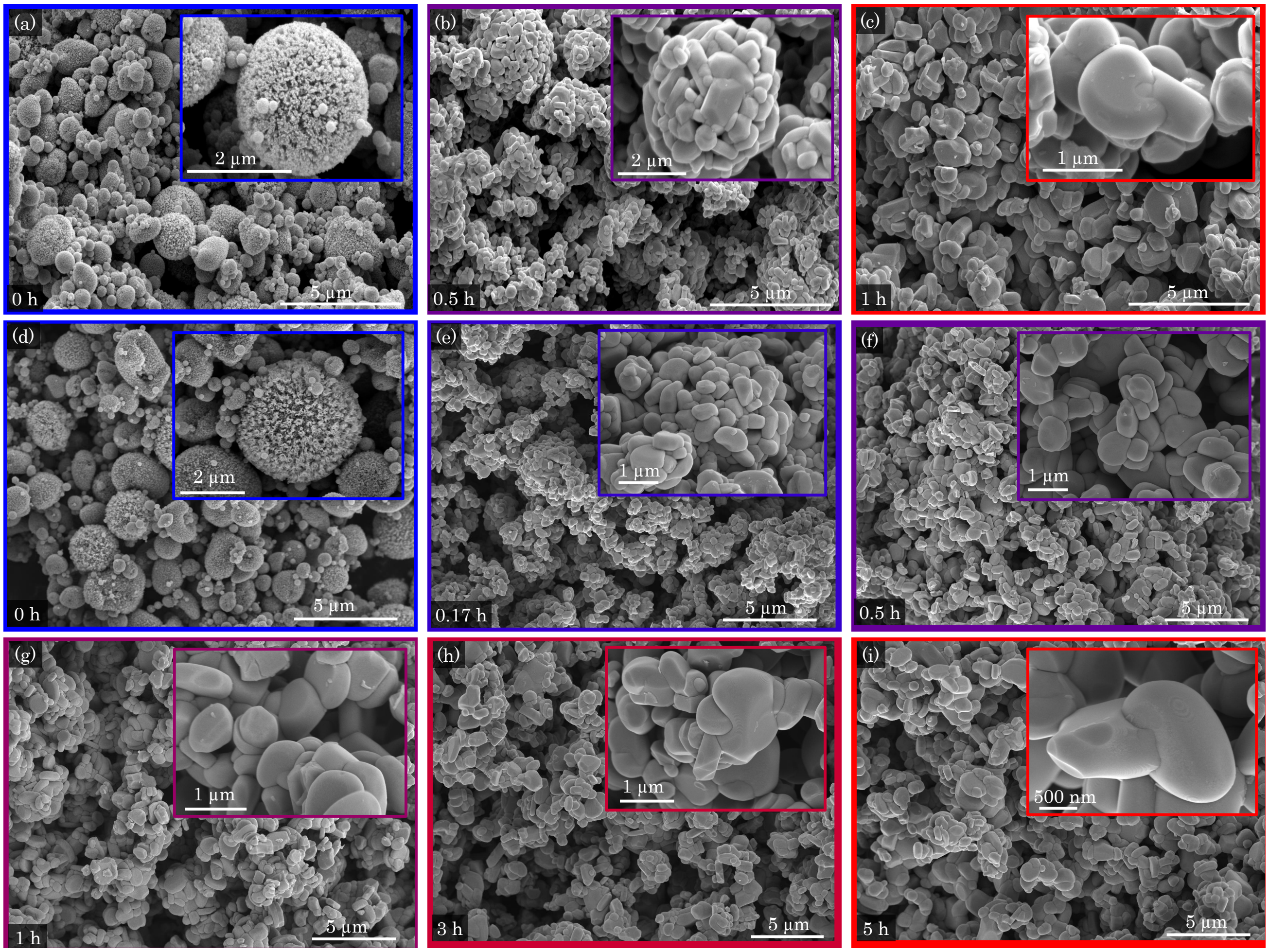

Figure S7: SEM images of LCO particles synthesized from nitrate precursors at (a-c) 800 °C and (d-i) 900 °C, followed by annealing at 775 °C for various durations.

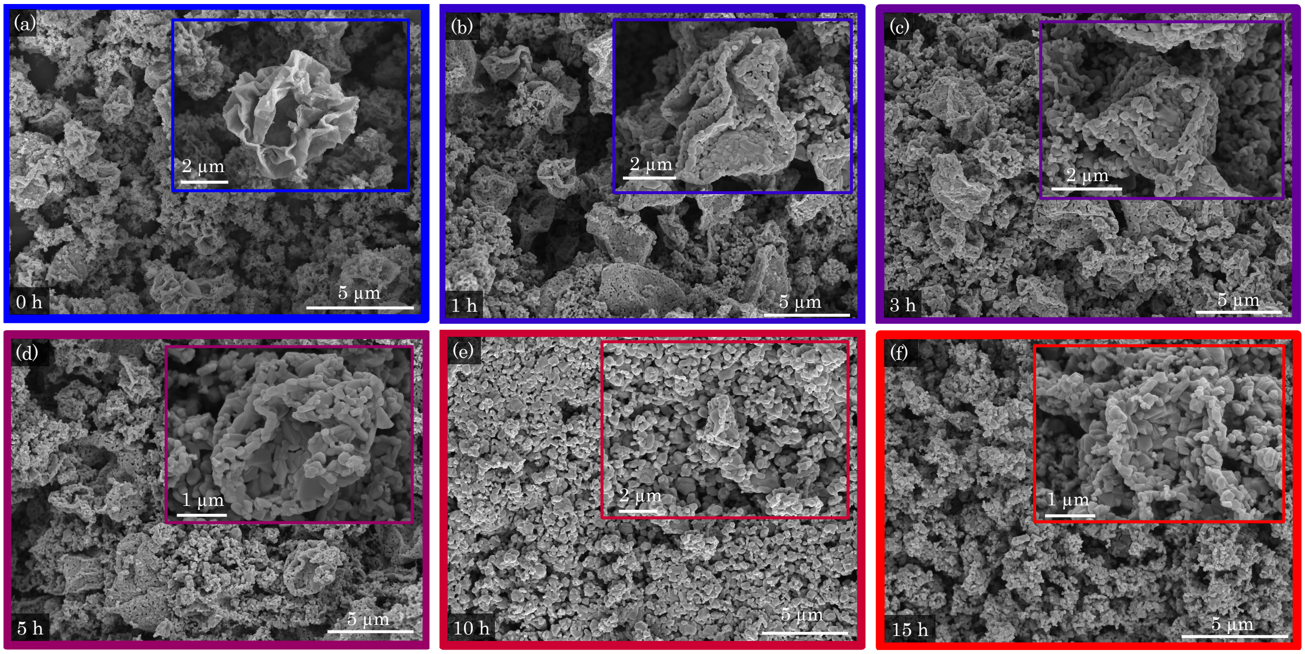

Figure S8: SEM images of LCO particles synthesized from acetate precursors at 900 °C and annealed at 750 °C for (a) 0 h, (b) 1 h, (c) 3 h, (d) 5 h, (e) 10 h, and (f) 15 h.

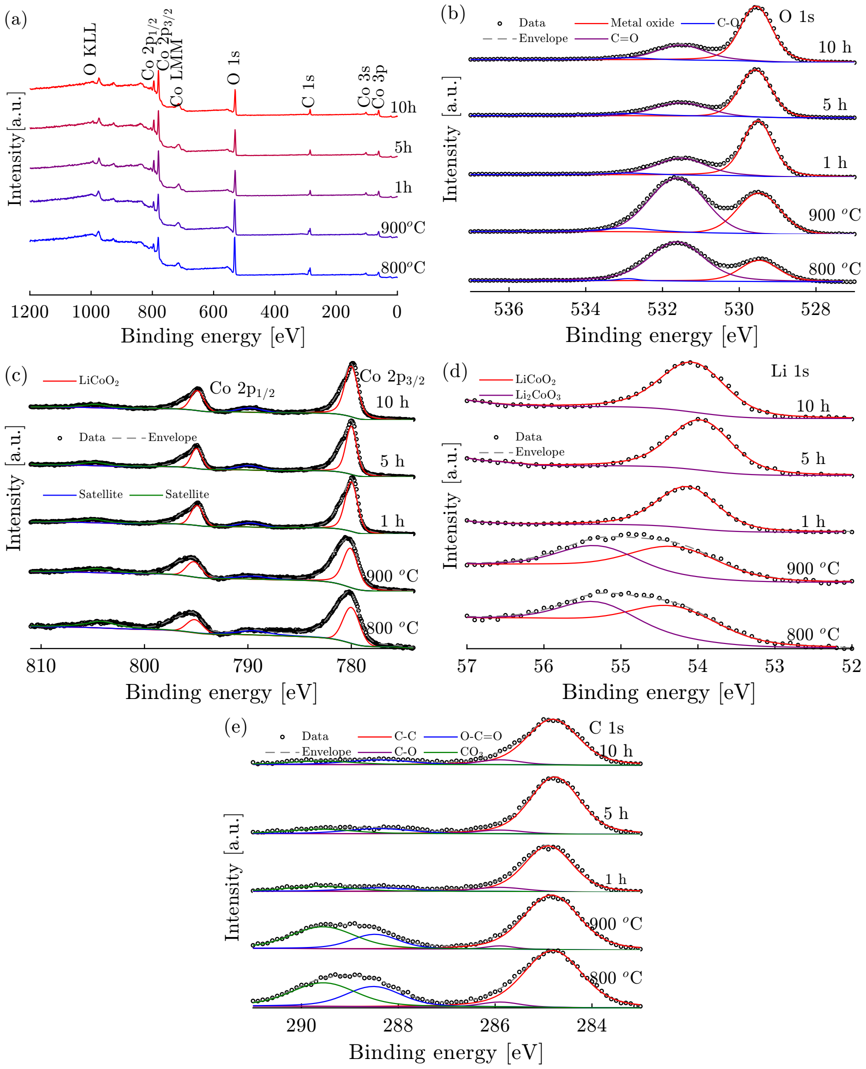

Figure S9: XPS analysis of LCO cathode materials prepared from acetate precursor. The figure displays (a) XPS survey scans, as well as spectra for (b) O 1s, (c) Co 2p, (d) Li 1s, and (e) C 1s for LCO cathode materials synthesized acetate precursor. Additionally, it includes data for two different synthesis temperatures and the annealed particles synthesized at the higher temperature.

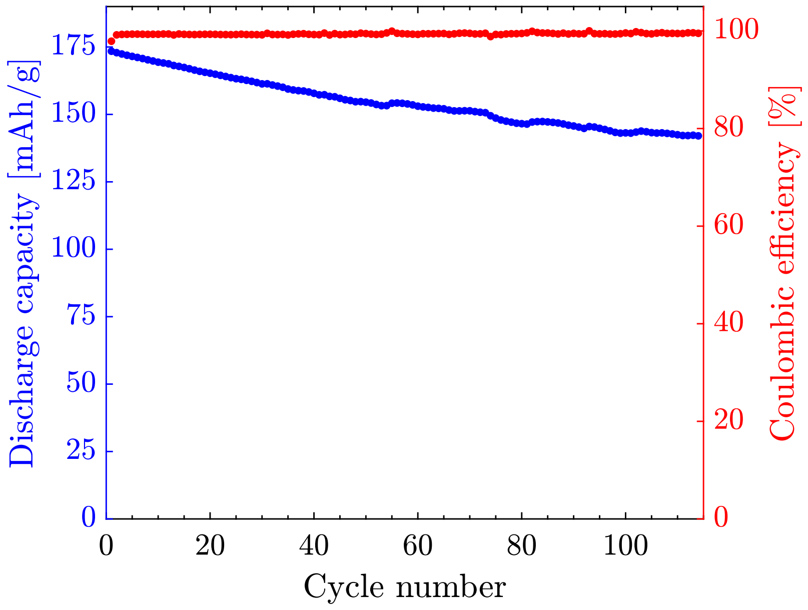

Figure S10: The cycling performance assessment over 115 cycles of LCO particles synthesized from nitrate precursors at 800 °C and annealed at 775 °C for 1 hour . The experiments were conducted using a 90:5:5 ratio of active material:carbon: binder and 20 μl of electrolyte.

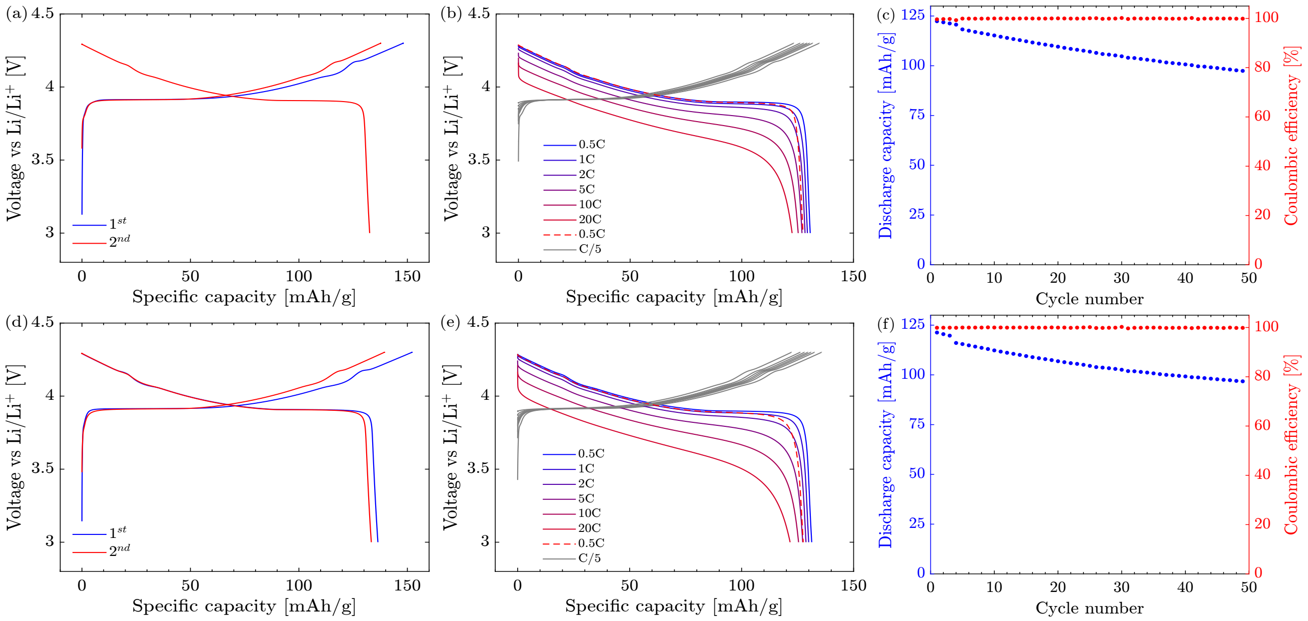

Figure S11: Electrochemical performance evaluation of LCO particles from acetate precursors. Synthesized at 900 °C and annealed at 750 °C for (a-c) 3 hours and (d-f) 15 hours, this includes (a,d) initial charge and discharge curves at 0.1C, (b,e) rate performance, and (c,f) cycling performance. The experiments were conducted using an 80:10:10 ratio of active material:carbon: binder and 80 μl of electrolyte.

References

(1) Rivas-Murias, B.; Salgueiriño, V. Thermodynamic CoO–Co3O4 Crossover Using Raman Spectroscopy in Magnetic Octahedron-Shaped Nanocrystals. *J. Raman Spectrosc.* **2017**, *48*, 837–841.

(2) Kang, S. G.; Kang, S. Y.; Ryu, K. S.; Chang, S. H. Electrochemical and Structural Properties of HT-LiCoO2 and LT-LiCoO2 Prepared by the Citrate Sol-Gel Method. *Solid State Ionics* **1999**, *120*, 155–161.
